# Surgical intra- and extra-articular anterior cruciate ligament reconstruction: a meta-analysis

**DOI:** 10.1186/s12891-020-03438-4

**Published:** 2020-06-30

**Authors:** Xiangyun Cheng, Fanxiao Liu, Dongsheng Zhou, Alexander C. Paulus

**Affiliations:** 1 Department of Orthopedic Surgery, Physical Medicine and Rehabilitation, University Hospital of Munich, Ludwig-Maximilians-University, Campus Großhadern, Marchioninistraße 15, 81377 Munich, Germany; 2 Department of Orthopaedics, Shandong Provincial Hospital affiliated to Shandong University, No.324, Road Jing Wu Wei Qi, Jinan, 250021 Shandong China

**Keywords:** Anterior cruciate ligament reconstruction, Extra-articular procedure, Pivot shift, Laxity measurements, Clinical outcomes

## Abstract

**Background:**

It is still controversial whether the combination of anterior cruciate ligament (ACL) reconstruction and extra-articular reconstruction (EAR) have good clinical efficacy. This meta-analysis aims systematically to compare the clinical effectiveness of ACL reconstruction and combined reconstruction.

**Methods:**

Electronic databases, including Medline/PubMed, Embase and the Cochrane Library, were systematically searched to identify targeted studies. A meta-analysis were performed to pool the outcome estimates of interest, such as the Lysholm, International Knee Documentation Committee (IKDC) and Tegner scores and the results from the KT-1000/2000 arthrometer test, the Lachman test and the pivot shift test.

**Results:**

Twelve studies involving 1146 knees were identified. Compared with single ACL reconstruction, combined reconstruction had better results for a pivot shift of grade 1 (relative ratio [RR] = 0.88, 95% CI: 0.83–0.94) and grade 2 (RR = 0.95, 95% CI: 0.91–0.99) rather than grade 3 (RR = 0.98, 95% CI: 0.90–1.06) and no statistically significant difference for both Lachman grade 1 (RR = 0.96, 95% CI: 0.89–1.05) and grade 2 (RR = 0.96, 95% CI: 0.90–1.03). Combined reconstruction resulted in significant improvements on the instrumented joint laxity test when considering a failure standard of more than 5 mm (a side-to-side arthrometric difference) (RR = 0.94, 95% CI: 0.89–0.98) rather than 3 mm (RR = 0.94, 95% CI: 0.86–1.03). Moreover, combined reconstruction increased the IKDC score at the 12-month (weighted mean difference [WMD] = − 6.38, 95% CI: − 9.66 to − 3.10), 24-month (WMD = − 5.60, 95% CI: − 8.54 to − 2.66) and 36-month follow-ups (WMD = − 4.71, 95% CI: − 7.59 to − 1.83) and the Tegner score at the 36-month follow-up (WMD = − 0.53, 95% CI: − 0.97 to − 0.09), but it did not increase the Lysholm score at the 36-month follow-up (WMD = − 0.84, 95% CI: − 2.02 to 0.34).

**Conclusion:**

With the advances in reconstruction techniques, combined reconstructions were found to be effective in improving rotational stability and to lead to good functional scores. However, obviously, the combined reconstruction technique is more time-consuming and requires an additional incision, which is not suitable for all ACL-deficient patients. Therefore, programs should be personalized and customized for the specific situation of each patient.

## Background

Anterior cruciate ligament (ACL) injuries, which account for approximately 60% of all knee injuries in pivoting sports, plague millions of sports participants [1, 2]. The ACL does not heal on its own when torn, and surgical reconstruction is the standard treatment [3, 4]. Although several surgical techniques for ACL reconstruction have been developed to be less invasive and more effective for patients, the optimal ACL reconstruction technique is still a highly demanding clinical issue in orthopaedic research [5]. Anatomic single-bundle (SB) ACL reconstruction, which only focuses on the reconstruction of the anteromedial bundle (AM), is commonly successful in limiting anterior tibial translation but may be deficient in controlling combined rotatory loads [6]; the failure rate ranging from 11 to 30%, was reported in the literature as being associated with persistent rotator instability [7]. Anatomic double-bundle surgery (DB), in which the AM and posterolateral bundles (PL) are reconstructed, may achieve a better restoration of the kinematic character of a normal knee than SB reconstruction, especially in terms of the rotational stability [8]. However, DB techniques cannot be performed in some ACL injured patients, because after synthetically considering the height and width of the fossa intercondyloidea and the ACL footprint, sometimes these structures cannot meet the requirements of reconstruction [9, 10]. Additionally, the enlarged risk of complications and the surgical complexity of DB reconstructions have restricted its widespread use [10].

With the aim of improving rotational control in ACL-deficient knees, SB reconstruction combined with extra-articular reconstruction (EAR) was popular since the beginning of modern ACL surgery [11]. However, EAR has been mostly given up since 1989 because some senior authors indicated that extra-articular techniques may be biomechanically reasonable but that they did not yield any improvement in clinical results and were even associated with a higher risk of degenerative osteoarthritis in both the patellofemoral and tibiofemoral compartments [12, 13]. Some researchers considered that at that time, open techniques, the exclusive use of bone-patellar tendon-bone and a very long period of immobilization with a brace or cast after the operation may have seriously affected the surgical results of combined reconstruction [11]. In recent years, the rapid development of arthroscopically assisted techniques, the various choices of intra-articular grafts and accelerated standard rehabilitation have resulted in a radical change in reconstruction techniques [14]. Additionally, an increasing number of researchers have a better understanding of the anterolateral structures of the knee [15]. After reviewing radiological images, up to 10% of ACL-deficient patients had a Segond fracture, which is a bony avulsion of the anterolateral ligament (ALL) [16]. As such, based on the recent renewed awareness of the anterolateral knee structures, a series of improved combined surgical techniques have been used by some research groups to improve joint function and rotational stability [17, 18].

Undoubtedly, there is still some controversy regarding the anterolateral knee structures [19, 20]. In terms of ALL, although the tibial insertion has been consistently described as being between Gerdy’s tubercle and the fibula, the femoral insertion and even its impact on rotational laxity relative to other anterolateral structures, are still a topic of some debate [15]. Despite some controversy, for patients with a high risk of graft laxity and failure, some recently published in vivo studies have tended to favour extra-articular tenodesis or reconstruction procedures combined with ACL reconstruction [17, 21]. However, several studies demonstrated that intra-articular plus additional anterolateral reinforcement procedures did not restore normal joint laxity and even over-constrain the lateral compartment [22, 23]. Compared with isolated intra-articular reconstruction, one systematic review published in 2015 indicated that combined intra- and extra-articular reconstruction (combined reconstruction) provided marginally improved knee stability and comparable failure rates, but no difference in patient-reported functional scores [24]. However, this meta-analysis only included a total of 8 studies, among which 4 studies were published in French, 1 study was published in the 1990s and 1 study compared combined reconstructions with double bundle ACL reconstruction, and these studies probably do not fully reflect recent advances. To the knowledge of the authors, no new meta-analysis on this topic has been published since 2015, yet multiple high-quality studies have been published, most of which have used relatively updated techniques and have had strict indications for lateral extra-articular procedures [10, 11, 17, 18, 21, 22, 25]. Therefore, an updated meta-analysis is warranted to determine if the new data and the improved combined techniques have a positive impact on the ACL-deficient knee compared with isolated intra-articular reconstruction.

Therefore, a systematic review and meta-analysis of clinical trials was conducted to compare single ACL reconstruction with combined reconstruction. The primary objective of this study was to determine whether adding an extra-articular reconstruction led to: 1) increased antero-posterior stability measured by the Lachman examination and a KT 1000/2000 arthrometer; (2) increased rotational stability measured by the pivot shift examination; and (3) better functional scores measured by the IKDC evaluation, Tegner score and Lysholm score. The hypothesis was that in terms of the outcome measures mentioned above, combined reconstructions have better results than single ACL reconstruction.

## Methods

The checklist of the Preferred Reporting Items for Systematic Reviews and Meta-Analyses (PRISMA) statement was followed in the conduction of this meta-analysis [26].

### Data sources and search strategy

Two independent investigators searched PubMed, Embase and the Cochrane Library (including Epub Ahead of Print) for titles from inception to September 24, 2019, with an iterative process using a combination of keywords and mesh terms: “anterior cruciate ligament”, “ACL”, “anterior cruciate ligament reconstruction”, “isolated intra-articular reconstruction” AND “anterolateral ligament reconstruction”, “ALL”, “knee extra-articular reconstruction”, “ACL combined reconstruction”, “extra-articular tenodesis” or “ACL with lateral tenodesis reconstruction”. The purpose, research question, and eligibility criteria for the search were determined a priori. The syntax, spelling, and general search strategy is presented in **Supplementary Table** **1**. Additionally, the reference lists of related articles (reviews, meta-analyses and included studies) comparing the efficacy of single ACL reconstruction with combined reconstruction were carefully screened to retrieve additional eligible studies not identified by electronic database searching.

### Study screening and selection

The included studies met all of the following criteria: 1) clinical studies comparing the efficacy of single ACL reconstruction with combined reconstruction; 2) participants consisting of patients with an ACL tear clinically diagnosed by imaging methods or arthroscopy; 3) sufficient data provided to calculate outcomes estimates of interests; 4) randomized/quasi-randomized/cluster controlled clinical trials and retrospective/prospective cohort studies; and 5) surgery to reconstruct the ACL is carried out via an arthroscopy.

The exclusion criteria were as follows: 1) reviews or meta-analyses; 2) animal or biomechanical studies; 3) anatomical study trials; 4) surgical guidelines or protocols about combined reconstructions; 5) studies without sufficient data to obtain endpoint outcomes of interest; 6) studies not describing the postoperative effectiveness of combined reconstructions; and 7) expert opinions, poster of abstracts, comments, letters and editorials because of their lack of data and methodology description.

Two investigators performed a blind systematic screening in duplicate. To maximize the sensitivity of the screen, disagreements at the title and abstract stages were resolved by automatic inclusion, whereas discrepancies at the full-text stage were resolved by consensus with input from a senior third investigator. We first removed redundant and unrelated records by screening titles and abstracts. Then, the full texts of the remainders were downloaded to confirm their eligibility based on the above criteria.

### Data extraction

The following information was collected from all included articles into a pre-designed Microsoft Excel spreadsheet (Version 2013, Microsoft, Redmond, WA, USA) independently by two blind investigators: first author’s family name, year of publication, region, study design, inclusion interval of patients, number of patients, demographic and clinical characteristics of participants (age and sex), number of knees, follow-up, and evaluation endpoint outcomes of interest (the pivot shift test, the Lachman test, the arthrometric KT-1000/2000 evaluation, the IKDC subjective score, the Tegner score and the Lysholm score). Data extraction from all included studies was completed in tandem by two independent investigators. The spreadsheets were combined, and each investigator checked a random selection of the other’s entries for quality control. Any discrepancies were resolved by consensus.

### Quality assessment

The methodological index for non-randomized studies (MINORS) is a valid tool designed to assess the methodological quality of non-randomized controlled studies. Meanwhile, the methodological quality of randomized studies was evaluated according to the Cochrane Handbook for Systematic Reviews of Interventions, which has five items for bias assessment including performance bias, detection bias, attrition bias, reporting bias, and other biases. The review of methodological quality was conducted in duplicate, blindly by two investigators. Any discrepancies were resolved by consensus.

### Statistical analysis

Inter- investigator agreement was calculated at each stage of the search, the screening, and the quality assessment of the included studies with a Kappa (κ) statistic. Agreement was categorized a priori as follows: 0.20 or less (poor agreement), 0.21–0.40 (fair agreement), 0.41–0.60 (moderate agreement), 0.61–0.80 (substantial agreement) and 0.81–0.99 (almost perfect agreement) [27]. A meta-analysis was implemented to conduct the quantitative analysis and produce forest plots using STATA 12.0 Version (V. 12.0, StataCorp, College Station, TX). A two-tailed *p* value < 0.05 indicated statistical significance. For continuous data, preference was given to analysing the results with the weighted mean difference (WMD) and the related 95% confidence intervals (CIs). For dichotomous data, the evaluation parameters of interest were assessed using relative ratios (RR) as well as related 95% CIs. Subgroup analyses were performed to explore the source of heterogeneity, which was assessed using *I*^*2*^. A sensitivity analysis was conducted by omitting studies with the maximum or minimum sample or non-randomized controlled trials; subgroup analysis was implemented according to two factors, including the study design (retrospective or prospective) and style of combined reconstruction (ACLR+ALLR), and the publication bias was determined using the Egger test to evaluate the stability of the results.

## Results

### Study inclusion and exclusion

**Fig. 1:**
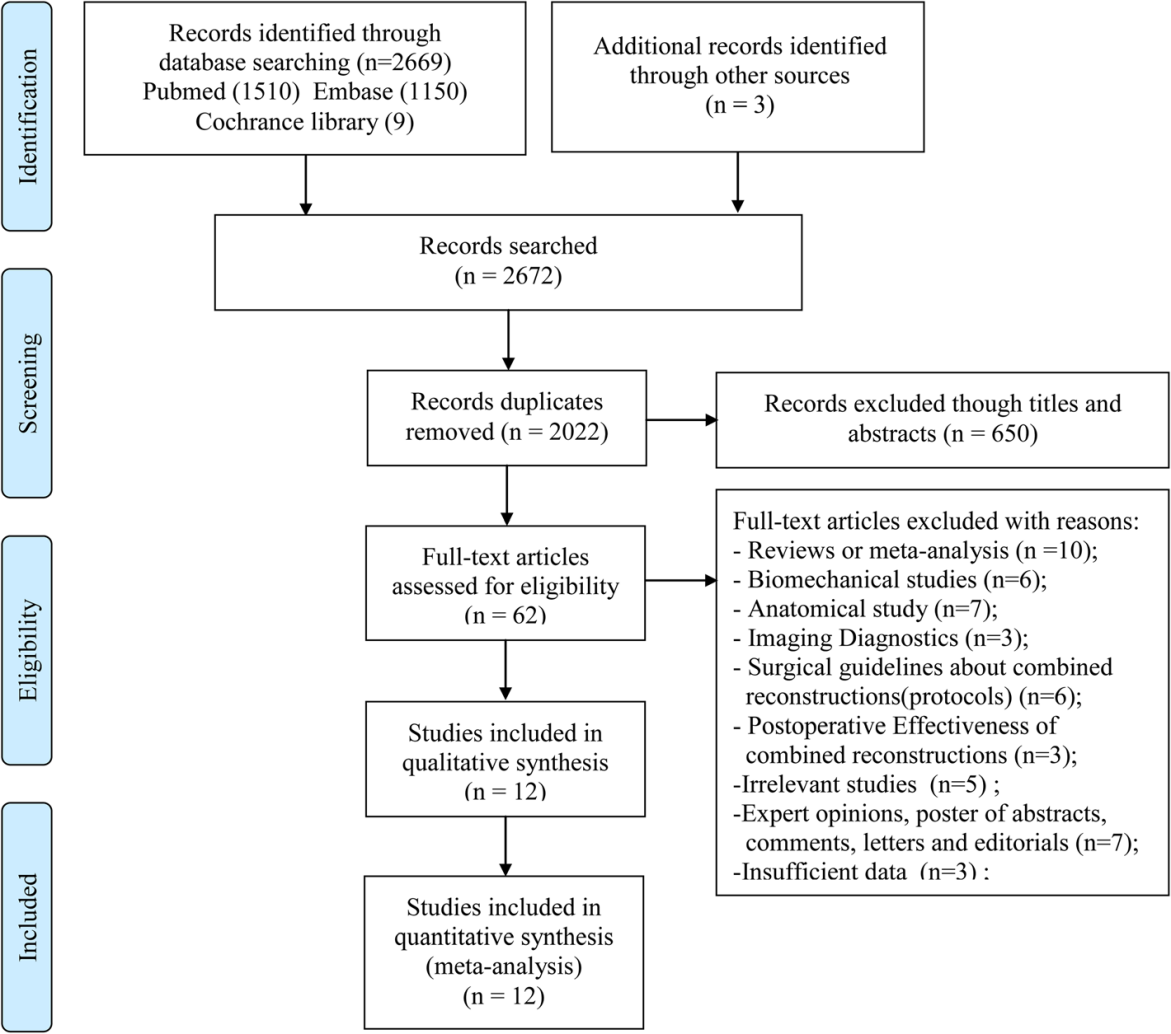
Flow diagram of the literature search and selection process

A total of 2669 records were screened in the electronic database search process with another 3 additional studies retrieved from screening the reference lists of related articles. After 650 duplicate studies were excluded, and 1960 studies excluded by reading titles and abstracts, 62 studies were downloaded and carefully checked by reading the full texts. Finally, a total of 12 studies [10, 11, 17, 18, 21–23, 25, 28–31] with 1037 patients involving 1146 knees were considered to be qualified for the quantitative analysis. The inclusion process and the reasons for exclusion are depicted in Fig. 1.

### Search results and included participants

The baseline methodological and procedural characteristics of the selected studies and the demographic data of enrolled participants are listed in Table 1. All included studies were published during 2001–2019 in English. The sample sizes of these studies ranged from 20 to 502. With regard to the design of the study, 8 studies [10, 22, 23, 25, 28–31] described prospective cohort studies, and 4 studies [11, 17, 18, 21] were retrospective cohort studies. In total, 5 studies [10, 17, 18, 21, 25] involved combined reconstructions of ACL and ALL. Main information of combined reconstruction technique used in each included studies are provided in Table 2.

**Table 1:** Main characteristics of the included studies

Author	Country	Groups of reconstruction	Person	M/F	Knee	Age	Time before surgery (months)	Inclusioninterval	Follow-up(range, months)	Studydesign	Grafts used in ACLR	LOE	Meniscus Injury (medial /lateral/both)
Lee, 2019 [21]	Korea	ACLR	45	34/11	45	27.3 ± 7.6	NA	3.2011–1.2013	41.5 ± 8.2	R	Gracilis tendon allograft (fresh frozen)	3	24
													25
		ACLR+ALLR	42	33/9	42	26.8 ± 6.1		2.2013–7.2014	38.2 ± 6.9				
Helito, 2018 [17]	Brazil	ACLR	68	59/9	68	33.9 ± 6.1	14 (12–30.5)	1.2011–6.2012	26 (24–29)	R	Gracilis and semitendinosus tendons	3	27
		ACLR+ALLR	33	30/3	33	33.1 ± 8.8	15 (13–18)	2014–2015	25 (24–28)		Gracilis and semitendinosus tendons		13
Imbert, 2017 [22]	Italy	ACLR	32	NA	32	NA	NA	NA	duringsurgery	P	Autologous semitendinosus and gracilis tendons	3	NA
		ACLR+EAR	32	NA	32	NA					Autologous semitendinosus and gracilis tendons		
Ibrahim, 2017 [25]	Kuwait	ACLR	50	50/0	50	26 (21–32)	3 (2.0–4.6)	1.2014–6.2014	27 (25–30)	P	Gracilis and semitendinosus tendons	2	9/7/3
		ACLR+ALLR	53	53/0	53	26 (20–30)	3 (2.0–4.4)		27 (25–30)				10/8/4
Sonnery-Cottet, 2017 [18]	France	ACLR	176	116/60	176	23.5 ± 4	4.5 ± 6.2	1.2012–5.2014	41 ± 6 7.0	R	Autogenous hamstring tendons	2	39/28/14
													55/36/34
		ACLR+ALLR	221	152/69	221	21.8 ± 4	5.3 ± 9.0		35.4 ± 8.4		Autogenous hamstring tendons		34/13/17
		ACLR	105	96/9	105	22.1 ± 3.7	6 ± 15.2		39.2 ± 8.8		Bone-patellar tendon-bone graft		
Zhang, 2016 [10]	China	SB-ACLR	20	13/7	20	22.3 ± 5.3	12.3 ± 4.3	7.2012–7.2015	3/6/12	P	Semitendinosus and gracilis tendons	2	NA
		DB-ACLR	20	14/6	20	28.3 ± 6.1	14.2 ± 4.6						
		SB-ACLR+ALLR	20	12/8	20	26.3 ± 6.8	16.3 ± 3.6						
Ferretti, 2016 [11]	Italy	ACLR	71	51/20	71	27.3 (18–50)	4	1.2002–12.2003	125 (121–128)	R	Semitendinosus and gracilis tendons	3	49
													41
		ACLR+EAR	68	56/12	68	25.7 (18–46)			126 (122–130)				
Trichine, 2014 [29]	Algeria	ACLR	52	52/0	60	27.7 ± 4.75	37.78	5.2007–7.2010	23.4 (6–45)	P	Patellar tendon	2	28/23
		ACLR+EAR	55	55/0	60	28.6 ± 4.69	35.48		24.5 (6–63)				14/16
Vadalà, 2013 [30]	Italy	ACLR	28	0/28	28	28 (15–40)	NA	1.2005–12.2006	43.1 (36–50)	P	Semitendinosus and gracilis tendons	3	11
		ACLR+EAR	27	0/27	27	26 (15–40)			45.2 (38–50)				
Monaco, 2007 [28]	Italy	ACLR	10	10/0	10	27 (17–40)	36 (6–72)	4.2006–5.2006	duringsurgery	P	Semitendinosus and gracilis tendons	3	10
		ACLR+EAR	10	10/0	10	27 (17–40)							
Zaffagnini, 2006 [31]	Italy	ACLR	25	15/0	25	31.3 (26–49)	6 (1–12)	1998–2003	60	P	Semitendinosus and gracilis tendons	2	NA
		ACLR	25	16/9	25	30.5 (22–47)					Bone-patellar tendon-bone graft		
		ACLR+EAR	25	18/7	25	26.7 (15–44)					Semitendinosus and gracilis tendons		
Anderson, 2001 [23]	USA	ACLR	35	23/12	35	20.1 (14–38)	0.5–3	1991–1993	35.9 ± 11.7	P	Semitendinosus and gracilis tendons	2	NA
		ACLR	35	23/12	35	23.6 (14–44)			34.6 ± 11.4		Bone-patellar tendon-bone graft		
		ACLR+EAR	35	22/13	35	22 (14–40)			35.7 ± 12.1		Semitendinosus and gracilis tendons		

*ACLR* anterior cruciate ligament reconstruction; *ALLR* anatomic anterolateral ligament reconstruction; *EAR* extra-articular reconstruction; *M* male; *F* female; *P* prospective; *R* retrospective; *LOE* level of evidence; *NA* not available

**Table 2:** Main information of combined reconstruction in the included studies

Study	Style of reconstruction	Grafts used in EAR	Graft fixation	Tibial Insertion	Femoral Insertion/Procedures
Lee, 2019 [21]	ALLR	A gracilis tendon allograft (fresh frozen)	7-mm biointerference screw (Matryx)	The center between the Gerdy tubercle and fibular head	Proximal and posterior to the lateral epicondyle
Helito, 2018 [17]	ALLR	Semitendinosus and gracilis tendons	Metal anchors/the iliotibial band is sutured	The center between the Gerdy tubercle and fibular head	The posterior aspect, the lateral epicondyle
Imbert, 2017 [22]	EAR	Semitendinosus and gracilis tendons	Interference screws	A tibial margin position against the posterior aspect of the Gerdy tubercle.	A femoral position 1 cm proximal and posterior to the lateral epicondyle
Ibrahim, 2017 [25]	ALLR	Semitendinosus and gracilis tendons	BioIntrafix interference screw	The center between the Gerdy tubercle and fibular head	The lateral femoral epicondyle; proximal and anterior to the lateral collateral ligament
Sonnery-Cottet, 2017 [18]	ALLR	Autogenous hamstring tendons	Bio-Interference screw	1 cm distal to the joint line: one just posterior to the Gerdy tubercle and the second one just anterior to the fibula head	Back proximally to the femur
Zhang, 2016 [10]	ALLR	Semitendinosus and gracilis tendons	The interference screw	At the position beyond the joint line 0.8–1.0 cm with equal distance to the Gerdy tubercle and fibular head.	At the prominence of the lateral femoral epicondyle, slightlyanterior to the origin of the lateral collateral ligament
Ferretti, 2016 [11]	MacIntosh modified Coker-Arnold procedure	Iliotibial band	Sutured under tension with periosteal absorbable stitches	The Gerdy tubercle	A portion of the iliotibial band is detached proximally, reflected and passed under the lateral collateral ligament, and sutured under tension with periosteal stitches to Gerdy tubercle, while the tibia is kept in maximum external rotation
Trichine, 2014 [29]	Extra-articular ilio-tibial band tenodesis	Iliotibial band	Interference screw/ n° 0 absorbable suture	The Gerdy tubercle	Isometric point of the lateral femoral condyle
Vadalà, 2013 [30]	MacIntosh modified Coker-Arnold procedure	Iliotibial band	#0 Vycril suture	The Gerdy tubercle	A portion of the iliotibial band is detached proximally, reflected and passed under the lateral collateral ligament, and sutured under tension with periosteal stitches to Gerdy tubercle, while the tibia is kept in maximum external rotation
Monaco, 2007 [28]	MacIntosh modified Coker-Arnold procedure	Iliotibial band	Periosteal stitches	The Gerdy tubercle	A portion of the iliotibial band is detached proximally, reflected and passed under the lateral collateral ligament, and sutured under tension with periosteal stitches to Gerdy tubercle, while the tibia is kept in maximum external rotation
Zaffagnini, 2006 [31]	EAR	Semitendinosus and gracilis tendons	A single staple	The Gerdy tubercle	In the cortical bone of the femur at the end of the lateral condyle
Anderson, 2001 [23]	A Losee extra-articular iliotibial band tenodesis	Iliotibial band	A whipstitch of 1–0 nonabsorbable material	The Gerdy tubercle	The lateral femoral condyle

*ALLR* anatomic anterolateral ligament reconstruction; *EAR* extra-articular reconstruction

Eight studies [10, 11, 17, 18, 21, 22, 28, 30] described non-randomized controlled trials with a mean MINORS score of 15.90 ± 1.46 (range, 14–18). Four studies [23, 25, 29, 31] assessed using the Cochrane handbook were described as randomized controlled trials, of which two studies [23, 29] had a high reporting bias, three studies [23, 25, 31] had an unclear bias of performance bias and four studies [22, 23, 29, 31] had a low risk of selection bias. The results of the quality assessment are presented in **Supplementary Table** **2****and Supplementary Fig.****1**. Substantial agreement amongst investigators was achieved at each stage: title (κ = 0.73, 95% CI: 0.69 to 0.78), abstract (κ = 0.87, 95% CI: 0.82 to 0.93), and full-text (κ = 0.86, 95% CI: 0.77 to 0.96), and there was substantial agreement on the quality assessment of the included studies (κ = 0.86, 95% CI: 0.79 to 0.93).

### Pivot shift test

**Fig. 2:**
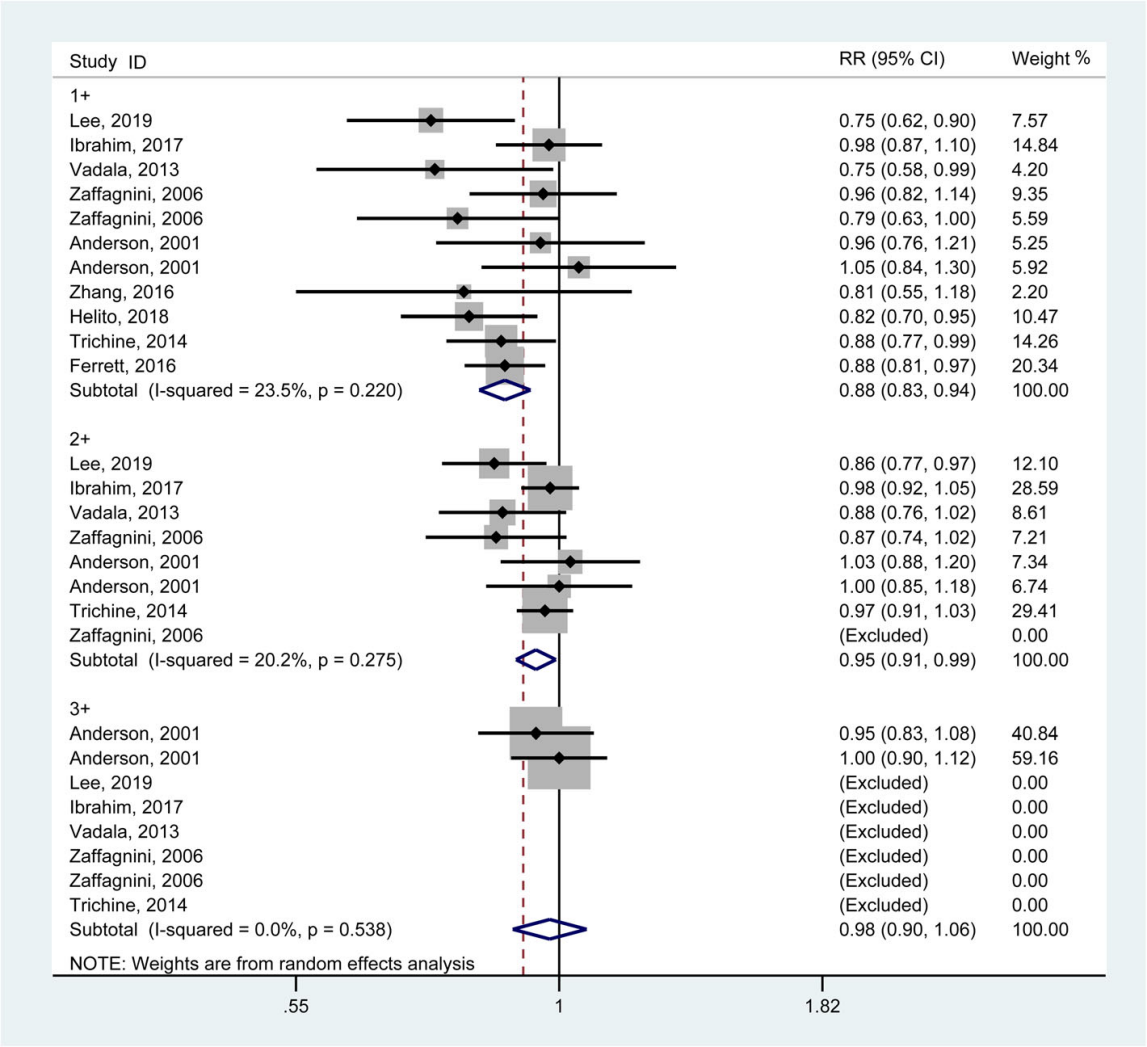
Forest plot comparing the pivot shift test outcomes between single ACL reconstruction and combined reconstructions. (RR, relative ratio; CI, confidence interval)

The pivot shift test, which is the standard way to evaluate rotatory instability, is used to evaluate knee rotatory laxity (graded as 0, 1+, 2+, 3+). In terms of the pivot shift test, the pooled results generated from 9 studies [10, 11, 17, 21, 23, 25, 29–31] involving 560 knees demonstrated that the patients with combined reconstructions had better results for grade 1 (RR = 0.88, 95% CI: 0.83–0.94) and grade 2 (RR = 0.95, 95% CI: 0.91–0.99) rather than for grade 3 (RR = 0.98, 95% CI: 0.90–1.06) (Fig. 2). The *I*^2^ statistics for heterogeneity were 23.5, 20.2 and 0.0% for grades 1, 2 and 3, respectively, which indicated no substantial heterogeneity among the included studies. The sensitivity analysis, which was conducted by omitting studies with the maximum or minimum sample or non-randomized controlled trials, and the subgroup analysis, which was implemented according to two factors, including style of combined reconstruction (ACL + ALL) (**Supplementary Fig.** **2**) and the study design (retrospective or prospective) revealed consistent trends.

### Lachman test

**Fig. 3:**
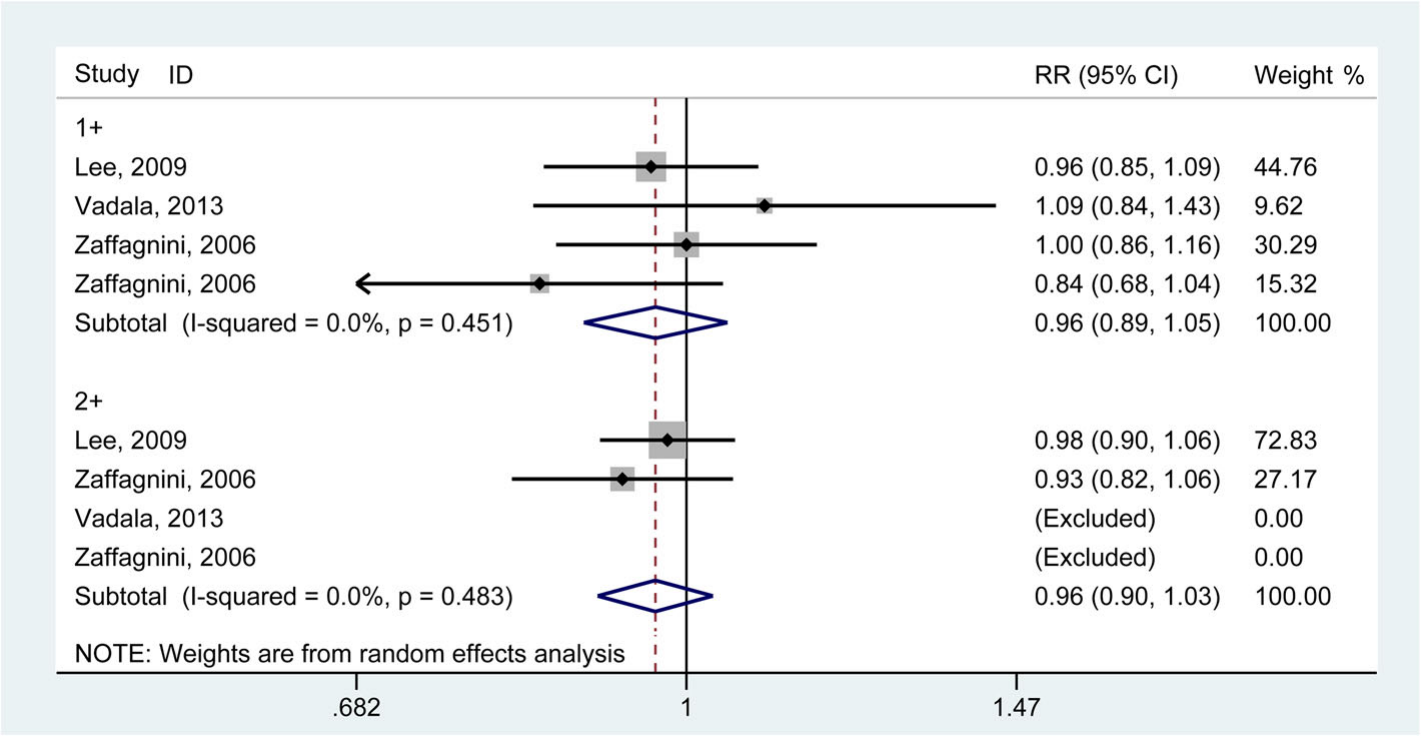
Forest plot comparing the Lachman test outcomes between single ACL reconstruction and combined reconstructions. (RR, relative ratio; CI, confidence interval)

Anterior knee laxity can be evaluated by the manual Lachman test (graded as 0, 1+, 2+, or 3+). The results generated from 3 studies [21, 30, 31] involving 191 knees demonstrated no significantly different for the Lachman test for grade 1 (RR = 0.96, 95% CI: 0.89–1.05) and grade 2 (RR = 0.96, 95% CI: 0.90–1.03) in both groups (Fig. 3). The *I*^2^ statistics for heterogeneity were 0.0 and 0.0% for grade 1 and 2, respectively, which indicated no substantial heterogeneity among the included studies. The sensitivity analysis and subgroup analysis revealed consistent trends (**Supplementary Fig.****3**).

### Instrumented knee laxity testing

**Fig. 4:**
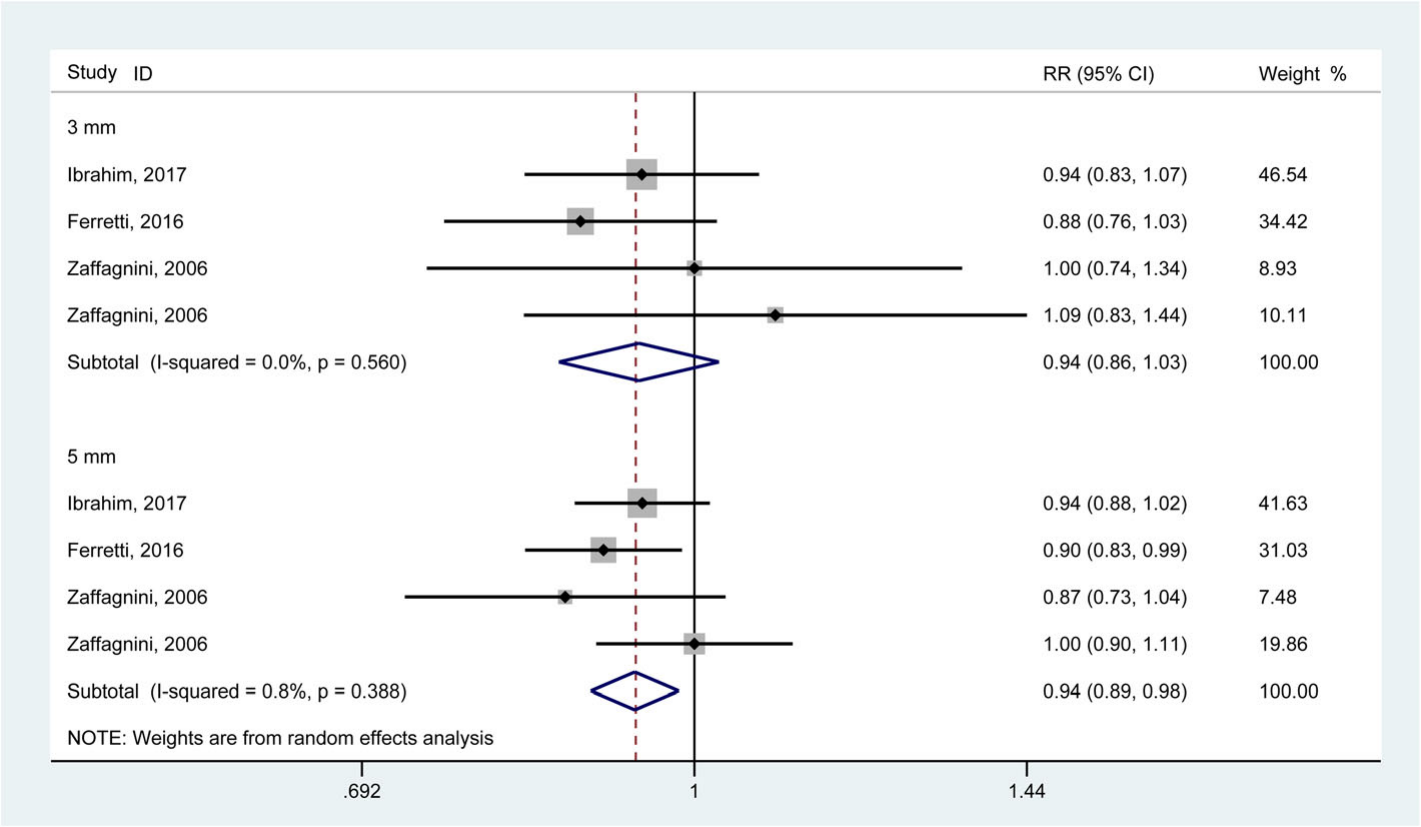
Forest plot comparing instrumented knee laxity testing between single ACL reconstruction and combined reconstructions using dichotomous data. (RR, relative ratio; CI, confidence interval)

**Fig. 5:**
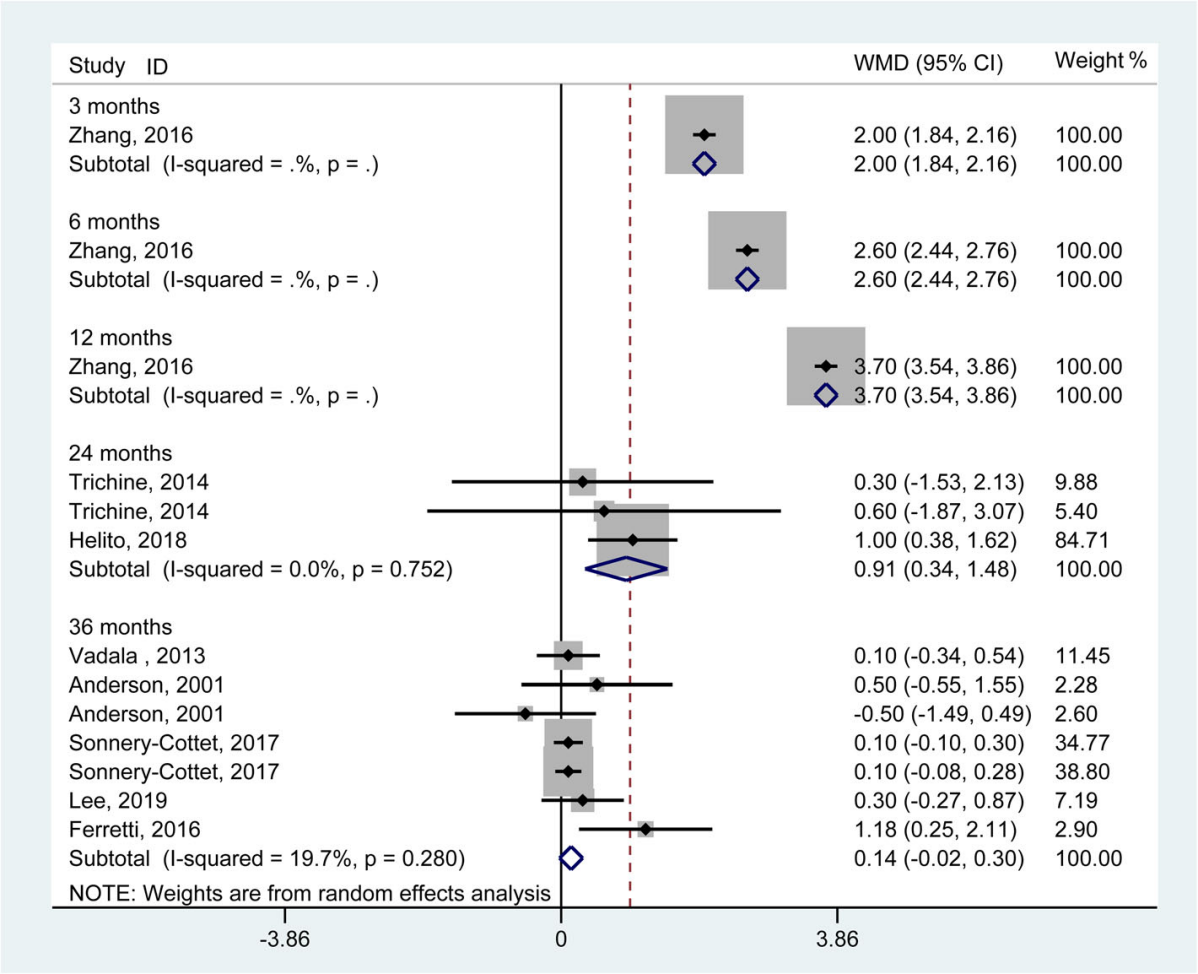
Forest plot comparing instrumented knee laxity testing between single ACL reconstruction and combined reconstructions using continuous data. (WMD, weighted mean difference; CI, confidence interval)

The KT-1000/2000 arthrometer test is usually used to evaluate the anterior translation of the tibia at a set pulling strength. The pooled results generated from 3 studies [11, 25, 31] involving 291 knees demonstrated significant improvements in antero-posterior stability when considering a failure standard of more than 5 mm (a side-to-side arthrometric difference) (RR = 0.94, 95% CI: 0.89–0.98) rather than 3 mm (RR = 0.94, 95% CI: 0.86–1.03) (Fig. 4). The *I*^2^ statistics for heterogeneity were 0.0 and 0.8% for more than 5 mm and 3 mm, respectively, which indicated no substantial heterogeneity among the included studies. For continuous data, the pooled results generated from 8 studies [10, 11, 17, 18, 21, 23, 29, 30] involving 985 knees demonstrated no significant outcomes in side-to-side arthrometric differences at the 36-month follow-up in both groups (WMD = 0.14, 95% CI: − 0.02 to 0.30, I^2^ for heterogeneity = 19.7%) (Fig. 5). The sensitivity analysis and subgroup analysis revealed consistent trends.

### Lysholm, IKDC and Tegner score

**Fig. 6:**
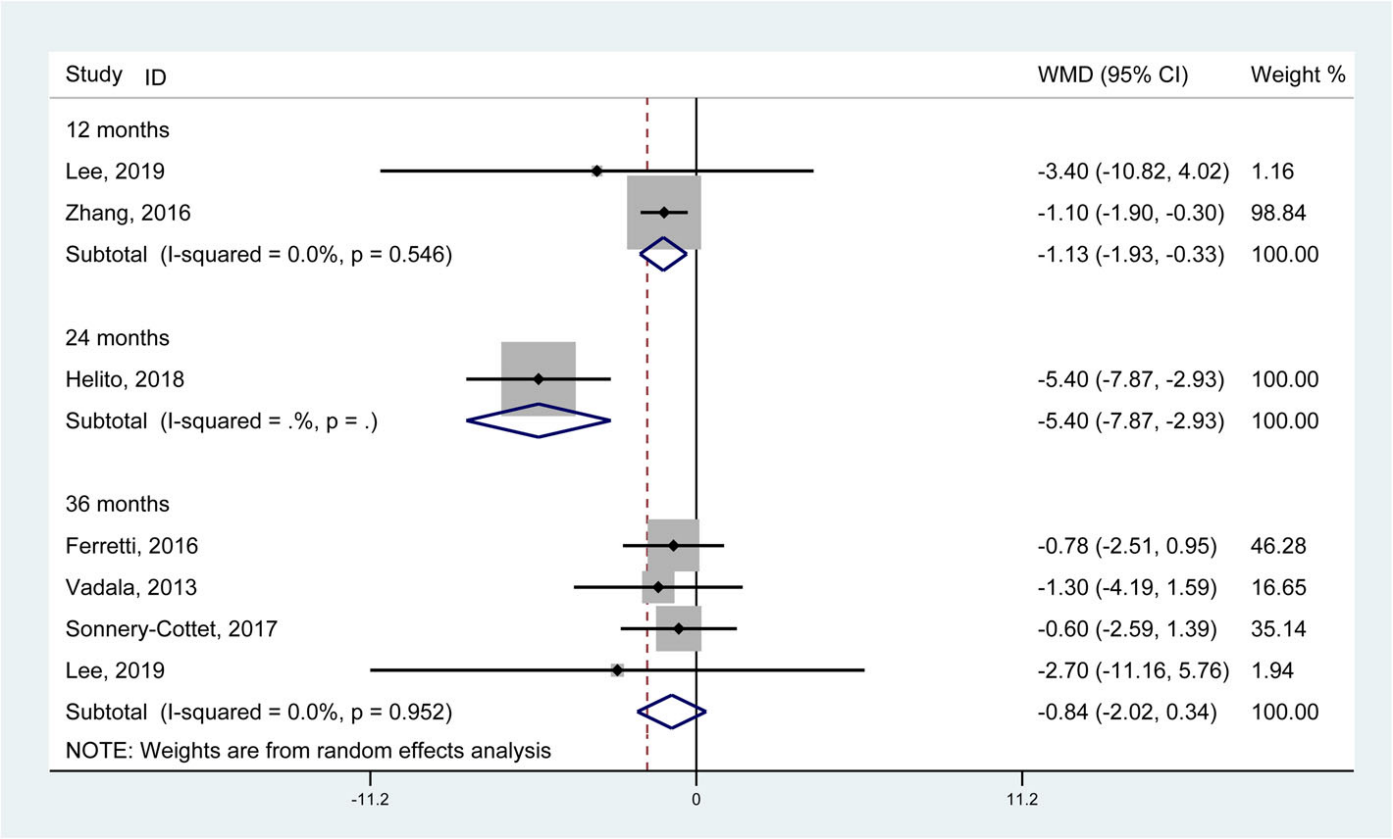
Forest plot comparing the Lysholm score between single ACL reconstruction and combined reconstructions. (WMD, weighted mean difference; CI, confidence interval)

In regard to the group of combined reconstructions, the results generated from 6 studies [10, 11, 17, 18, 21, 30] involving 944 knees confirmed a significant increase of Lysholm score at the 12- and 24-month (WMD = − 1.13, 95% CI: − 1.93 to − 0.33; − 5.40, 95% CI: − 7.87 to − 2.93) but not significant at the 36-month follow-ups (WMD = − 0.84, 95% CI: − 2.02 to 0.34) (Fig. 6).

**Fig. 7:**
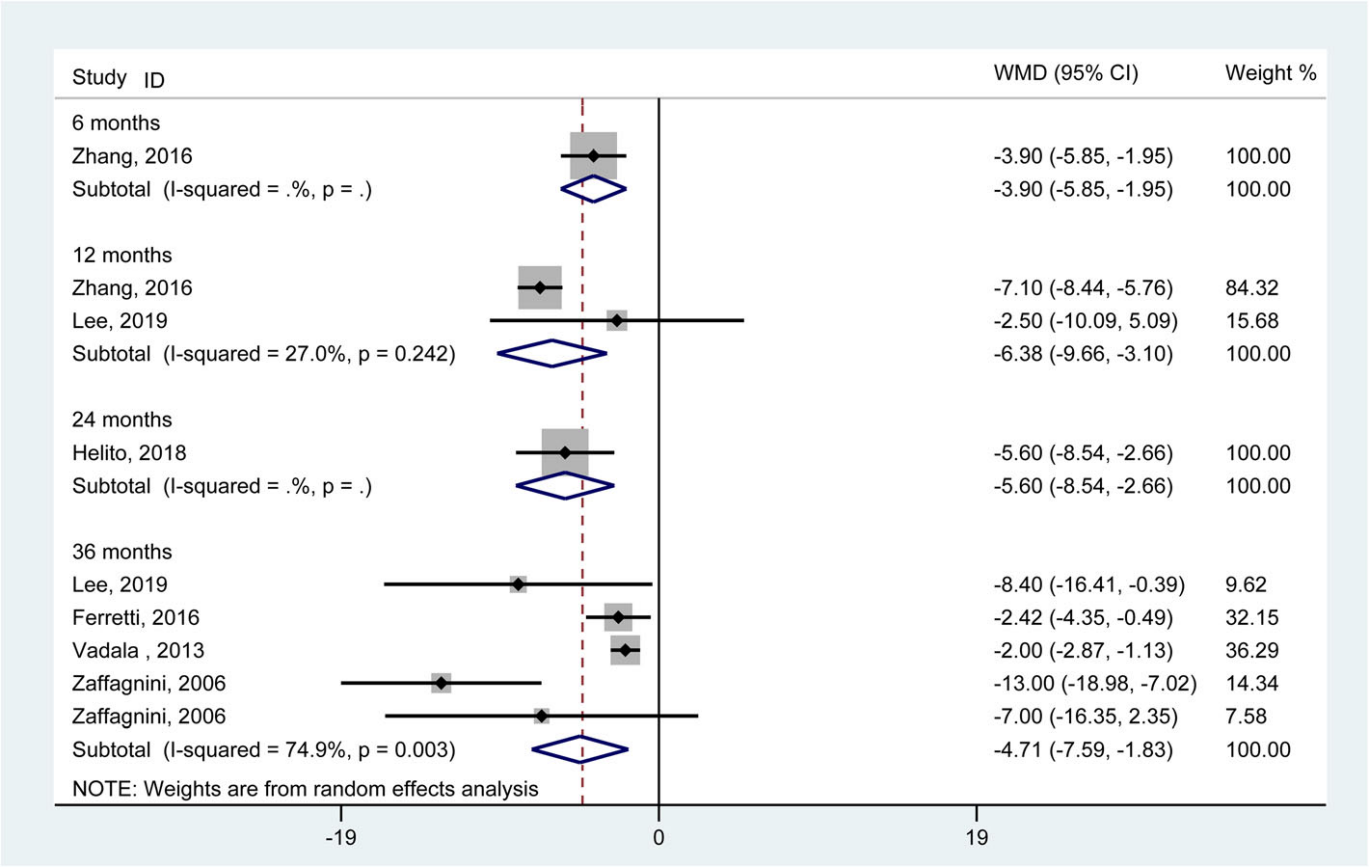
Forest plot comparing the IKDC score between single ACL reconstruction and combined reconstructions. (WMD, weighted mean difference; CI, confidence interval)

In relation to the postoperative IKDC subjective scores, the group of combined reconstructions revealed better results than the group of single ACL reconstruction at the 12-, 24- and 36-month follow-ups (WMD = − 6.38, 95% CI: − 9.66 to − 3.10; − 5.60, 95% CI: − 8.54 to − 2.66; − 4.71, 95% CI: − 7.59 to − 1.83) (Fig. 7). Both the *I*^2^ statistics for heterogeneity at the 12- and 24-month follow-ups were 0.0%, which indicated no substantial heterogeneity among the included studies; however, the *I*^2^ statistic for heterogeneity at the 36-month follow-up was 74.9%, which revealed substantial heterogeneity among the included studies. The subgroup analysis, which was implemented according to the style of combined reconstruction (ACL + ALL), revealed consistent trends. (**Supplementary Fig.** **4**). Additionally, the pooled results generated from 5 studies [10, 18, 21, 30, 31] involving 698 knees demonstrated a significant increase in the Tegner score at the 36-month follow-up (WMD = − 0.53, 95% CI: − 0.97 to − 0.09) (**Supplementary Fig.** **5**).

## Discussion

The initial hypothesis was accepted, except for in regard to the anteroposterior stability measured by the Lachman test. The pooled results revealed that patients with an ACL injury who underwent combined reconstructions had better rotational stability and improvements in functional outcome scores compared to patients who underwent isolated ACL reconstruction. However, there were no significant differences between the two groups regarding anteroposterior stability measured by the Lachman test in this meta-analysis.

Tears of the ACL can lead individuals to long absences from sports and can even result in permanent sports disability [32]. In the past few decades, ACL reconstruction, as the most effective treatment for serious ACL tears, has significantly improved knee functional outcomes and life quality in the majority of ACL-deficient patients [33]. However, persistent rotatory knee laxity is still a common finding after single bundle ACL reconstruction, and it can be a critical cause of the development of further articular injuries [20]. While the reasons behind rotatory instability of the knee are multifactorial, the impact of the anterolateral knee structures (including the anterolateral complex [ALC] and ALL) is significant [20]. After exploring the anterolateral structures of certain patients with acute ACL injury, some researchers indicated that 90% of ACL injuries were accompanied by injuries of the anterolateral structures [34]. Therefore, some patients may benefit from reinforcement of the anterolateral structures combined with ACL reconstruction.

Our pooled results for the pivot shift test confirmed that patients with combined reconstructions had better rotational stability than patients with an isolated single bundle ACL reconstruction, which demonstrated that extra-articular procedures can significantly contribute to rotatory knee stability. The anterolateral knee structures associated with an ACL injury were been described as early as 1879 [35, 36], but at that time, it was quite difficult to anatomically define the related anterolateral knee structures due to the complexity of lateral knee anatomy and the undeveloped dissection techniques. Therefore, during the early period, owing to the poor understanding of the anterolateral knee structures, there were many unsatisfactory surgical outcomes of extra-articular reconstruction [13, 37]. In recent years, many researchers have managed to better describe the anatomy of the anterolateral knee structures and to emphasize the role of some specific ligamentous structures (e.g., the anterolateral ligament) in controlling rotational instability of the knee and the role of reducing the pivot shift phenomenon [38, 39]. Based on these studies, surgical techniques for anatomic combined reconstruction have been advocated, with many promising preliminary results [40]. Many researchers have attempted to determine the reasons for extra-articular procedures controlling rotational instability. Ferretti et al. once revealed that extra-articular reconstructed tissues, close to the centre of rotation of the knee, had a longer lever arm for controlling rotation, and that it was far greater than that provided by a central intra-articular reconstruction [11]. In addition, several published biomechanical studies also indicated that in the ACL-deficient knee, the load-bearing ability of anterolateral knee structures (mainly referring to ALL) increased to approximately 3-fold in response to the pivot-shift test [41]. Therefore, mainly due to their ability to control rotatory laxity and to share loads with the ACL graft, extra-articular reconstructed structures improved rotational stability, and this was demonstrated by most of our included studies [10, 11, 17, 21, 28, 30, 31].

However, in contrast to other studies, two of the included articles in our study showed that there was no significant difference between combined reconstructions and isolated intra-articular reconstructions regarding the pivot shift test [23, 25]. One article was from Anderson et al. [23]; in their included group of combined reconstructions, 29 of 35 patients had a torn lateral or medial meniscus, and 21 of 29 injured menisci were partially excised, which may have interfered with rotational stability. Meniscal loss, especially lateral meniscal loss, plays a significant role in the manifestation of the pivot shift [20]. The other study was from Ibrahim et al. [25]; although their analysis did not reveal any statistically significant difference in the pivot shift test, a higher percentage of normal results was observed among the patients who underwent combined reconstructions. Therefore, regarding the overall effect on rotational stability, there was a very positive effect by the combined reconstruction techniques.

In terms of the Lachman test, no significant difference was found between the combined reconstruction and the isolated intra-articular reconstruction in this pooled study, which was consistent with many other clinical studies [18, 21, 30]. Based on many clinical studies, there was general agreement that single bundle ACL reconstruction could often achieve comparatively ideal antero-posterior stability after an ACL rupture [10, 29]. Therefore, because of the good control of antero-posterior stability by the intra-articular graft, additional extra-articular procedures did not seem to improve antero-posterior stability [42]. However, for the instrumented knee laxity test that detects anterior translation, better results were found in the combined reconstructions group. From the included studies, some patients who underwent isolated ACL reconstruction had anterior translations of more than 5 mm, but patients with combined constructions did not have this problem. Therefore, as the structure of secondary restraints, the extra-articular reconstructed graft probably plays an effective role in anterior tibial translation, especially in situations with strong pulling strength. Additionally, combined reconstruction is associated with a reduction in the rate of graft ruptures compared with isolated intra-articular reconstructed techniques [40, 43]. This finding can likely be attributed to load sharing of the EAR. One biomechanical study demonstrated that the load-bearing ability of the anterolateral structures in an ACL-intact knee was minimal in response to the simulated anterior drawer and Lachman tests [41, 43]. However, in the ACL-deficient knee, the load-bearing ability of the anterolateral structures significantly increased in response to the anterior drawer and Lachman tests [41].

In relation to the postoperative IKDC subjective score, the combined reconstruction group revealed better results than the isolated intra-articular reconstruction group at all follow-ups in our study. In terms of the Lysholm score and Tegner score, patients who underwent combined reconstruction also had higher scores at most of the follow-up periods. The results in our study were different from the previous meta-analysis published in 2015 [24], which had similar results in these patient-reported outcome scores between the two groups. For the patient-reported outcome scores mentioned above, our included studies were published mainly after 2015 [10, 11, 17, 18, 21], as such, the difference between the two meta-analyses indicated that combined reconstruction techniques may improve functional recovery with the development of related techniques.

In our pooled study, combined reconstructions showed quite effective results, however, there is still some controversy in choosing the ideal patients who would benefit from the extra-articular procedures. The combined reconstruction technique is more time-consuming and requires an additional incision, which is not suitable for all ACL-deficient patients, especially people who do not participate in pivot sports [20]. In addition, there were some concerns that extra-articular procedures could over-constrain the knee and consequently contribute to early lateral compartment degeneration [44]. Based on these considerations, all of the included studies in our meta-analysis had various strict indications for performing combined reconstructions, mainly including patients with high rotatory laxity [11, 25, 30], chronic ACL-deficiency patients (more than 1-year since injury) [28, 31] and high risk of graft rupture [17, 18, 21]. With the fast development of related techniques, we believe that a consensus about “ideal patients” will be reached in the near future.

Several limitations exist in this systemic review and meta-analysis. The main limitations of this study originate from the data pooled from the included articles. Randomized controlled trials (RCTs) and nRCTs were both included when comparing combined reconstructions and isolated ACL reconstruction, and having a greater caseload in the nRCTs especially may lead to bias. Nevertheless, the MINORS scores were acceptable when evaluating the quality of nRCTs. Additionally, because a full appreciation of ALL’s functional importance in normal and sport activities is still being established, in actual clinic work, various extra-articular reconstruction techniques are being used. Some techniques aim at re-tensioning and reinforcing the anterolateral capsule to reconstruct the ALL itself. Other techniques tend to perform anatomic ALL reconstruction based on a new understanding of anterolateral structures. The conclusion of the pooled analysis shows the development of extra-articular reconstructions, but not ALL reconstructions. The follow-up periods were diverse, and the results of each follow-up were processed to reduce heterogeneity; however, in some subgroups, enough data could not be obtained.

## Conclusion

With the advances in reconstruction techniques, combined reconstructions were found to be effective in improving rotational stability and to lead to good functional scores. However, obviously, the combined reconstruction technique is more time-consuming and requires an additional incision, which is not suitable for all ACL-deficient patients. Therefore, programs should be personalized and customized for the specific situation of each patient.

## Supplementary information

**Additional file 1.**

**Additional file 2.**

## Data Availability

All data analyzed during this study are included in this published article.

## Abbreviations

ACL: Anterior cruciate ligament
ALC: Anterolateral complex
ALL: Anterolateral ligament
AM: Anteromedial bundle
DB: Double-bundle
EAR: Extra-articular reconstruction
IKDC: International knee documentation committee
MINORS: Methodological index for non-randomized studies
RCTs: Randomized controlled trials
PL: Posterolateral bundle
RR: Relative ratio
PRIMA: Systematic reviews and meta-analyses statement
SB: Single-bundle
SMD: Standardized mean difference
WMD: Weighted mean difference
