# Supplementary material for: Surgical intra- and extra-articular anterior cruciate ligament reconstruction: a meta-analysis
Source: BMC Musculoskelet Disord. 2020 Jun 30;21:414. doi: 10.1186/s12891-020-03438-4 (PMC7325370; doi:10.1186/s12891-020-03438-4)
Supplement: Supplementary file 1 — Additional file 1. [file 12891_2020_3438_MOESM1_ESM.docx]

| **Supplementary Table 1: Search strategy** | | |
| --- | --- | --- |
| Pubmed | (anterior cruciate ligament? OR ACL? OR anterior cruciate ligament reconstruction? OR isolated intra-articular reconstruction?) AND (ALL? OR anterolateral ligament reconstruction? OR knee extra-articular reconstruction? OR ACL combined reconstruction? OR extra-articular tenodesis? OR ACL with lateral tenodesis reconstruction?) | 1510 |
| Embase | ('anterior cruciate ligament'/exp OR 'anterior cruciate ligament' OR (anterior AND cruciate AND ('ligament'/exp OR ligament)) OR 'acl'/exp OR acl OR 'anterior cruciate ligament reconstruction'/exp OR 'anterior cruciate ligament reconstruction' OR (anterior AND cruciate AND ('ligament'/exp OR ligament) AND ('reconstruction'/exp OR reconstruction)) OR 'isolated intra-articular reconstruction' OR (isolated AND 'intra articular' AND ('reconstruction'/exp OR reconstruction))) AND ('anterolateral ligament reconstruction'/exp OR 'anterolateral ligament reconstruction' OR (anterolateral AND ('ligament'/exp OR ligament) AND ('reconstruction'/exp OR reconstruction)) OR 'knee extra-articular reconstruction' OR (('knee'/exp OR knee) AND 'extra articular' AND ('reconstruction'/exp OR reconstruction)) OR 'acl combined reconstruction' OR (('acl'/exp OR acl) AND combined AND ('reconstruction'/exp OR reconstruction)) OR 'extra-articular tenodesis' OR ('extra articular' AND ('tenodesis'/exp OR tenodesis)) OR 'acl with lateral tenodesis reconstruction' OR (('acl'/exp OR acl) AND with AND lateral AND ('tenodesis'/exp OR tenodesis) AND ('reconstruction'/exp OR reconstruction))) | 1150 |
| Cochrane library | ((anterior cruciate ligament) or ACL or (anterior cruciate ligament reconstruction) or (isolated intra-articular reconstruction)) AND (ALL or (anterolateral ligament reconstruction) or (knee extra-articular reconstruction) or (ACL combined reconstruction) or (extra-articular tenodesis) or (ACL with lateral tenodesis reconstruction)) .af. | 11 |

| **Supplementary Table 2.** The results of MINORS score. | | | | | | |
| --- | --- | --- | --- | --- | --- | --- |
| Study | Country | No. of groups | Follow-up (months) | Study design | LOE | MINORS score |
| Lee, 2019 | Korea | 2 (ACLR/ACLR+ALLR) | 38.2 ± 8.2 | R | 3 | 18 |
| Helito, 2018 | Brazil | 2 (ACLR/ACLR+ALLR) | 25 (24-29) | R | 3 | 16 |
| Imbert, 2017 | Italy | 2 (ACLR/ACLR+EAR) | During surgery | P | 3 | 15 |
| Sonnery-Cottet, 2017 | France | 3 (ACLR/ACLR+ALLR) | 35 ± 8.4 | R | 2 | 17 |
| Zhang, 2016 | China | 3 (ACLR/ACLR+ALLR) | 3/6/12 | P | 2 | 16 |
| Ferretti, 2016 | Italy | 2 (ACLR/ACLR+ALLR) | 125 (121-128 ) | R | 3 | 17 |
| Vadala, 2012 | Italy | 2 (ACLR/ACLR+EAR) | 43.1 (36–50) | P | 3 | 14 |
| Monaco, 2007 | Italy | 2 (ACLR/ACLR+EAR) | During surgery | P | 3 | 14 |
| ACLR, anterior cruciate ligament reconstruction; ALLR, anatomic anterolateral ligament reconstruction; EAR, extra-articular reconstruction; P, prospective; R, retrospective; LOE, level of evidence; MINORS, methodological index for non-randomized studies. | | | | | | |


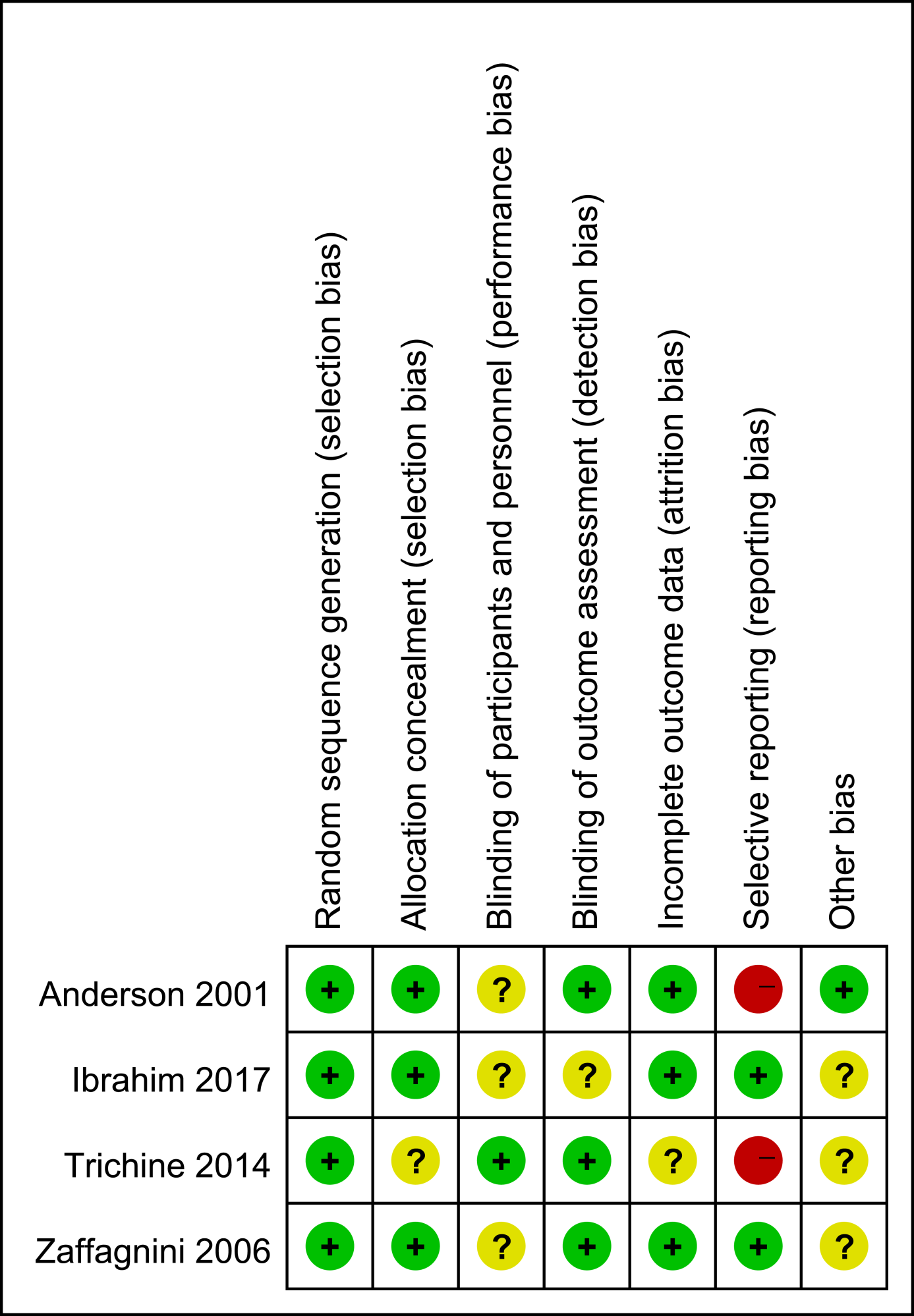


**Supplementary Fig. 1** The results of quality assessment of randomized studies.


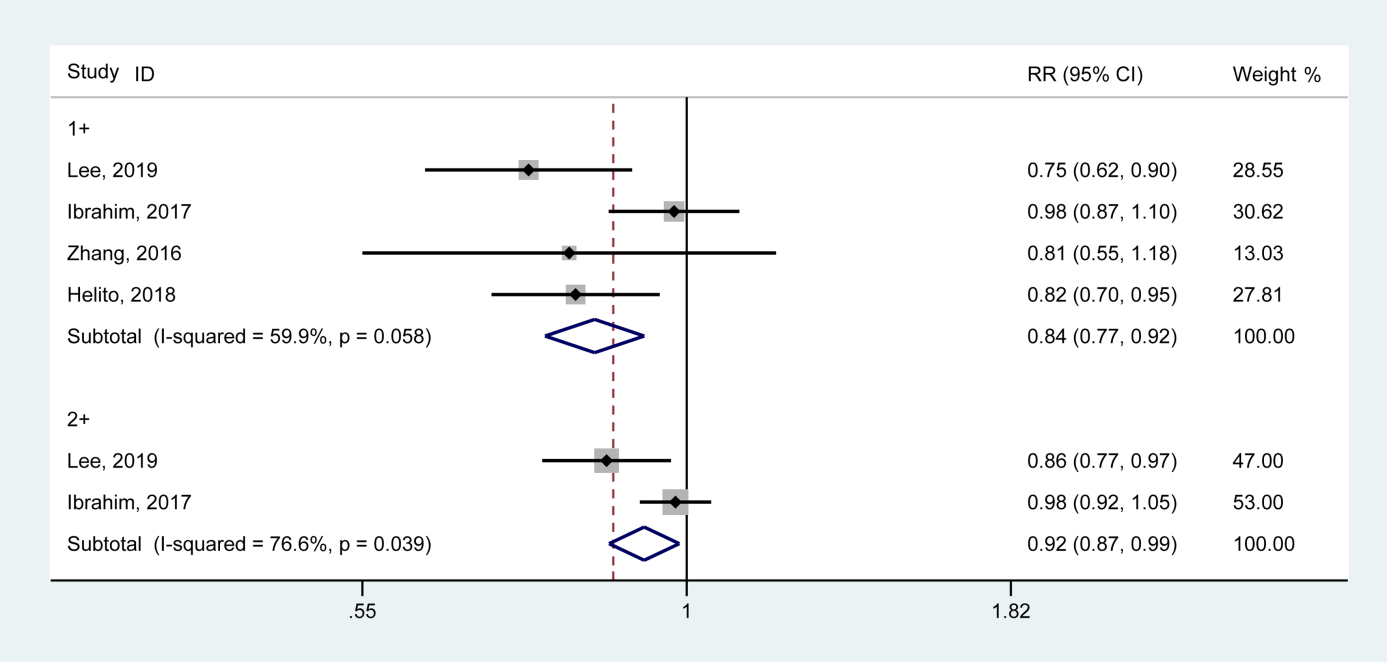


**Supplementary Fig. 2** Forest plot comparing the pivot shift test outcomes between single ACL reconstruction and ACL+ALL reconstructions. (ACL, anterior cruciate ligament; ALL, anterolateral ligament; WMD, weighted mean difference; CI, confidence interval)


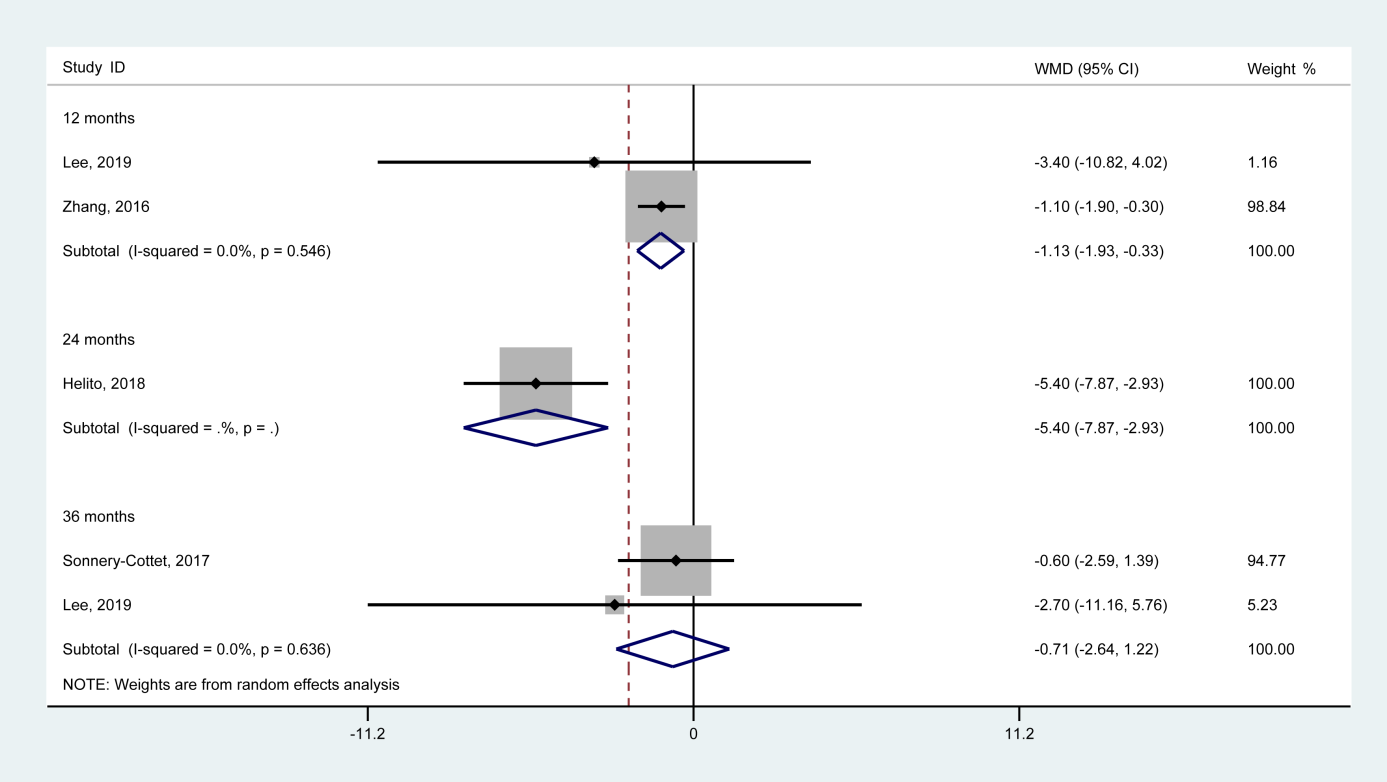


**Supplementary Fig. 3** Forest plot comparing the Lachman test outcomes between single ACL reconstruction and ACL+ALL reconstructions. (ACL, anterior cruciate ligament; ALL, anterolateral ligament; WMD, weighted mean difference; CI, confidence interval)


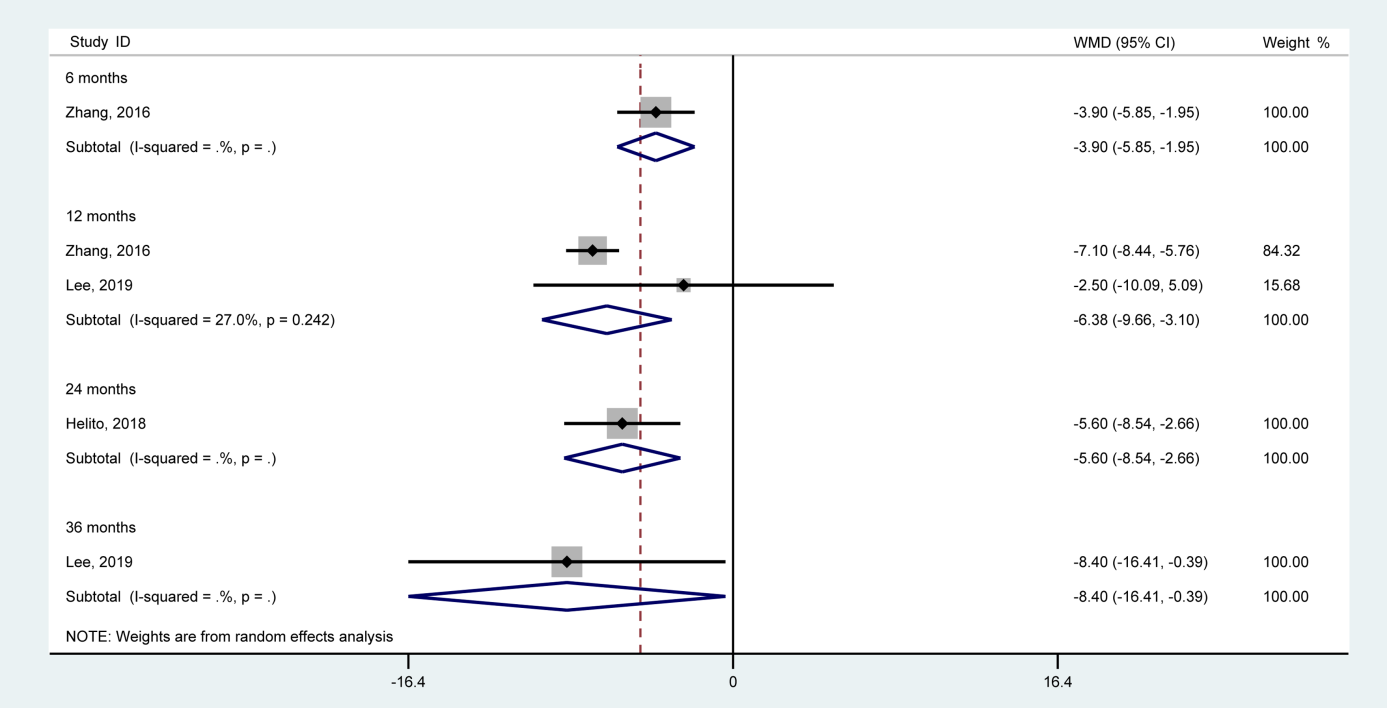


**Supplementary Fig. 4** Forest plot comparing the IKDC score between single ACL reconstruction and ACL+ALL reconstructions. (IKDC, international knee documentation committee; ACL, anterior cruciate ligament; ALL, anterolateral ligament; WMD, weighted mean difference; CI, confidence interval)


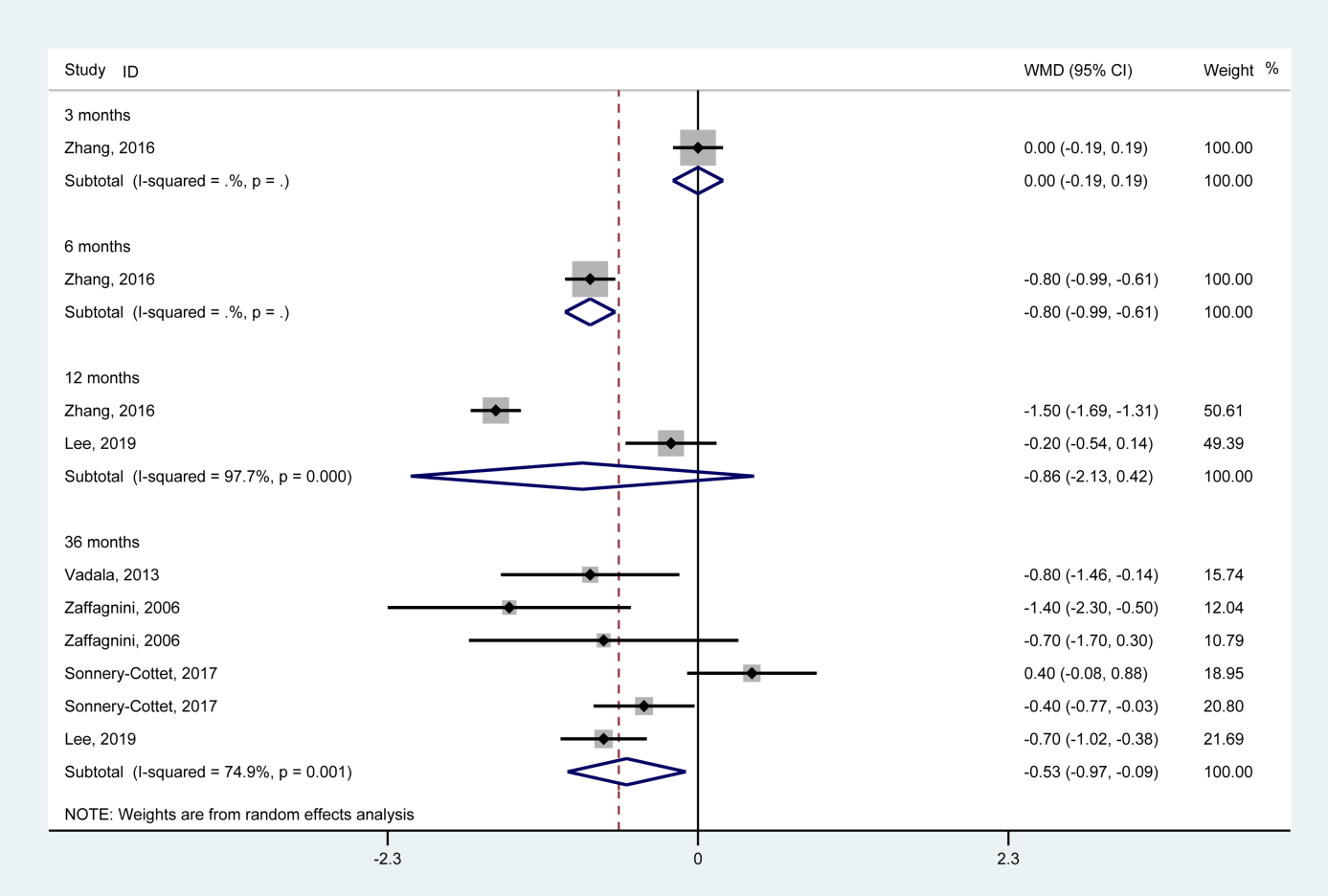


**Supplementary Fig. 5** Forest plot comparing Tegner score between single ACL reconstruction and combined reconstruction. (ACL, anterior cruciate ligament; WMD, weighted mean difference; CI, confidence interval)
